# Safety pharmacology and toxicology of a novel nitroimidazooxazole antitubercular agent in SD rat and Beagle dogs

**DOI:** 10.1186/s43556-026-00414-7

**Published:** 2026-03-02

**Authors:** Dandan Peng, Furong Qin, Minyang Fu, Ning Jiang, Manni Wang, Zhenling Wang, Xiawei Wei

**Affiliations:** 1 Laboratory of Aging Research and Cancer Drug Target, State Key Laboratory of Biotherapy and Cancer Center, National Clinical Research Center for Geriatrics, West China Hospital, Sichuan University, No. 17, Block 3, Southern Renmin Road, Chengdu, Sichuan 610041 People’s Republic of China; 2 Jumbo Drug Bank Co., Ltd., Chengdu, China

**Keywords:** JBD0131, Anti-tuberculosis, Preclinical safety, Toxicology, Pharmacokinetics

## Abstract

We developed JBD0131, a novel nitroimidazooxazole antitubercular agent, and conducted a comprehensive preclinical evaluation of its safety pharmacology, toxicology, and pharmacokinetics in SD rats and Beagle dogs. JBD0131 was well-tolerated in repeated-dose oral studies, with no treatment-related mortality or significant alterations in organ weights or significant alterations in organ-to-body weight ratios observed. The no-observed-adverse-effect level (NOAEL) was established at 480 mg/kg/day in rats and 300 mg/kg/day in female dogs. In male dogs, the NOAEL was determined to be 15 mg/kg/day, a discrepancy primarily attributed to a slight trend toward Corrected QT interval (QTc) prolongation at higher doses (60 and 300 mg/kg/day) to which males exhibited greater cardiovascular sensitivity. Pharmacokinetic analysis revealed dose-proportional systemic exposure with no accumulation of JBD0131. Although the metabolite DM131 showed moderate accumulation, it was identified as the amino-reduction detoxification product of JBD0131, a conversion that yields a more stable species and is supported by favorable clinical safety data. While Phase I clinical trials of JBD0131 have been reported, this preclinical study remains indispensable as it establishes the toxicological "ceiling" and defines safety margins through supra-therapeutic dosing. By identifying sex-specific sensitivities and clarifying metabolite safety, this work provides a critical scientific foundation for long-term clinical monitoring and risk assessment. Based on indirect comparisons with reported historical data for clinical agents such as bedaquiline and pretomanid, JBD0131 demonstrated a favorable preclinical safety profile in the models tested, supporting its continued development for multidrug-resistant tuberculosis.

**Supplementary Information:**

The online version contains supplementary material available at 10.1186/s43556-026-00414-7.

## Introduction

Tuberculosis (TB) remains one of the oldest but still most prevalent infectious diseases worldwide, posing a major threat to global public health. According to the World Health Organization (WHO), an estimated 10.8 million people developed TB in 2023, and 1.25 million deaths were attributed to the disease [1]. With the waning impact of the COVID-19 pandemic, TB may again become the leading cause of mortality from a single infectious agent. Importantly, TB is both preventable and curable, and global control efforts have saved nearly 79 million lives since 2000 [2].

Nevertheless, inappropriate use of anti-TB drugs—such as inadequate prescriptions, poor drug quality, or premature discontinuation of therapy—has led to the emergence of drug-resistant forms of TB. Multidrug-resistant TB (MDR-TB), defined by resistance to isoniazid and rifampicin, undermines the effectiveness of standard first-line therapy [3]. More alarmingly, extensively drug-resistant TB (XDR-TB) has emerged, characterized by resistance not only to first-line drugs but also to key second-line agents, including fluoroquinolones and aminoglycosides [4, 5]. Despite the availability of treatment regimens, MDR-TB continues to represent a pressing global health crisis, with only about 40% of patients accessing therapy in 2023 [2].

In recent years, the development of new therapeutics for MDR-TB has advanced considerably, with nitroimidazole-based compounds representing a particularly promising class [6]. These agents exhibit bactericidal activity under both aerobic and hypoxic conditions, making them effective against both replicating and dormant Mycobacterium tuberculosis. Thus, nitroimidazoles are considered important candidates for shortening treatment duration and improving clinical outcomes. Representative compounds such as delamanid and pretomanid have already reached clinical application or regulatory approval [7, 8]. However, their clinical utility is constrained by poor aqueous solubility, limited oral bioavailability, and adverse events such as peripheral neuropathy and anemia [9, 10]. Specifically, delamanid is associated with clinically significant QTc prolongation via hERG inhibition, while pretomanid has shown potential for testicular toxicity and peripheral neuropathy in preclinical models [11, 12]. These clinical and preclinical liabilities emphasize that potency alone is insufficient for successful translation, necessitating early and rigorous safety de-risking of new nitroimidazole candidates.

To address this gap, our group has developed a novel series of nitroimidazooxazole derivatives [13]. These compounds retain structural features of delamanid but compare favorably with the reported literature values for pretomanid in preclinical assays. Following systematic optimization, we identified JBD0131 as a lead candidate, which exhibited robust antimycobacterial activity in vitro [14]. While clinical Phase I and II evaluations of JBD0131 have recently been reported, these human studies primarily focus on therapeutic tolerability and pharmacokinetics in limited cohorts [15, 16]. Unlike clinical trials that are ethically constrained to therapeutic dose ranges, this preclinical package systematically explores the "toxicological ceiling" of JBD0131 through supra-therapeutic dosing. This allows for the calculation of exposure-based safety margins and the identification of subtle, sex-specific sensitivities—such as the QTc variations observed in male dogs—that are essential for informing the safety monitoring protocols of ongoing and future clinical investigations.

## Results

### JBD0131 shows good safety in SD rats

To assess the potential acute effects of JBD0131 on the central nervous and respiratory systems, a safety pharmacology study was conducted in SD rats. Single oral dose of JBD0131 at 50, 150, and 500 mg/kg were well tolerated in SD rats. Throughout the observation period, no mortality or treatment-related clinical abnormalities were detected. As detailed in Table S1, neurobehavioral assessments—including home-cage observations, handling/restraint responses, and open-field assessments—including fecal and urinary characteristics-showed no statistically significant differences compared with pre-dose values or vehicle controls (*P* > 0.05).

While minor fluctuations were noted in urination frequency, rearing counts, grip strength, and body temperature at isolated time points (Table S1). However, these changes were neither dose-dependent nor indicative of adverse toxicological effects (Table 1). Respiratory examinations, including respiratory rate, tidal volume, and minute ventilation, demonstrated no consistent deviations from control animals, indicating normal respiratory function (Table S2).

**Table 1:** Effects of oral gavage of JBD0131 on open field observation parameters in SD rats

Parameter	Time point	Control group		Vehicle Group		50 mg/kg Group		150 mg/kg Group		500 mg/kg Group	
		n	Mean ± SD	n	Mean ± SD	n	Mean ± SD	n	Mean ± SD	n	Mean ± SD
Fecal pellet count	Before administration (0 h)	10	1.5 ± 1.8	10	2.0 ± 1.6	10	2.4 ± 2.3	10	2.5 ± 2.4	10	3.1 ± 1.5
	8 h after administration	10	2.2 ± 1.9	10	2.7 ± 2.1	10	2.8 ± 3.2	10	1.6 ± 1.7	10	2.8 ± 2.1
	24 h after administration	10	2.9 ± 2.0	10	3.5 ± 1.8	10	2.1 ± 2.6	10	3.4 ± 2.5	10	3.3 ± 0.9
	48 h after administration	10	1.8 ± 1.3	10	2.8 ± 2.0	10	2.5 ± 1.6	10	2.6 ± 2.1	10	2.9 ± 2.4
Number of urinations	Before administration (0 h)	10	0.0 ± 0.0	10	0.8 ± 0.6 *	10	0.2 ± 0.4	10	0.9 ± 0.3 *	10	0.6 ± 0.5 *
	8 h after administration	10	0.8 ± 0.6 ▲	10	0.4 ± 0.7	10	0.4 ± 0.5	10	0.9 ± 0.3	10	0.3 ± 0.5
	24 h after administration	10	0.0 ± 0.0	10	1.2 ± 0.8 *	10	0.5 ± 0.5	10	0.9 ± 0.6 *	10	1.1 ± 0.6 *
	48 h after administration	10	0.4 ± 0.5 ▲	10	0.0 ± 0.0 ▲	10	0.9 ± 0.9	10	0.8 ± 0.4	10	0.2 ± 0.4
Number of rearing	Before administration (0 h)	10	6 ± 1	10	12 ± 4 *	10	10 ± 2	10	9 ± 4	10	9 ± 3
	8 h after administration	10	13 ± 2 ▲	10	10 ± 3	10	16 ± 5 ▲	10	12 ± 4	10	11 ± 4 ▲
	24 h after administration	10	7 ± 1	10	15 ± 3 ▲*	10	11 ± 5 *	10	7 ± 4	10	12 ± 2 ▲*
	48 h after administration	10	7 ± 2	10	9 ± 1 ▲	10	10 ± 2 *	10	7 ± 4	10	11 ± 3 *

^*^Compared with the control group, the difference between group means is statistically significant (*P* ≤ 0.05)

▲ Compared with the pre-administration time point, the difference in means is statistically significant (*P* ≤ 0.05)

Overall, single-dose oral administration of JBD0131 did not produce treatment-related effects on neurobehavioral activity, physiological function, or respiratory parameters in SD rats. These findings support a favorable acute safety profile and provide a basis for further preclinical development.

### JBD0131 shows good safety in Beagle dogs

To evaluate the cardiovascular impact of JBD0131, we monitored hemodynamic parameters in Beagle dogs, a standard non-rodent model for cardiovascular safety. Consistent with the rat study, Beagle dogs were assigned to three dose groups (15, 60, and 300 mg/kg), with solvent and saline controls. Throughout the dosing and observation periods, all dogs remained in good clinical condition, exhibited normal behavior, and showed no treatment-related adverse reactions.

**Fig. 1:**
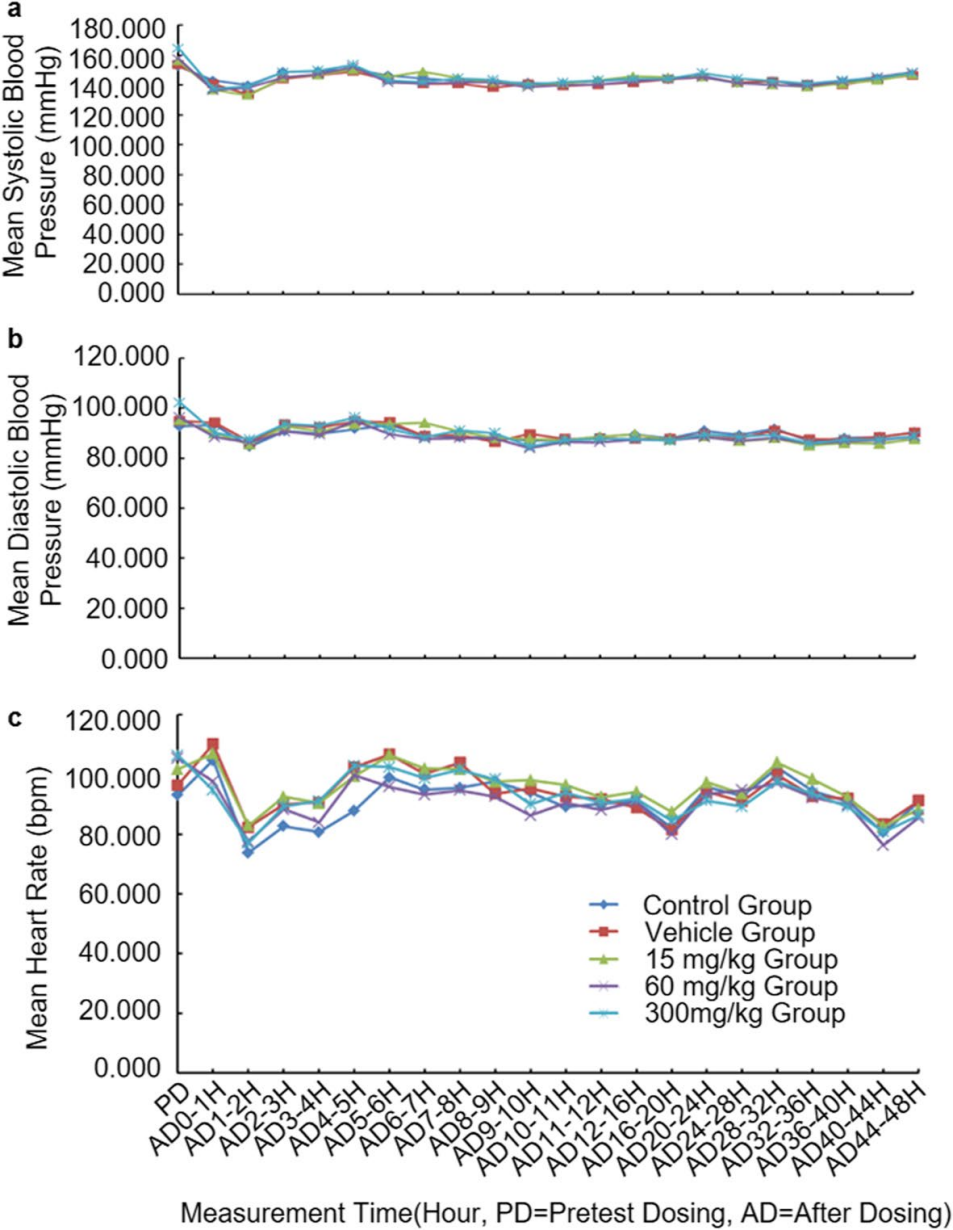
JBD0131 had no effects on the cardiovascular system in Beagle dogs. **a**-**c** Effects of JBD0131 on systolic blood pressure (**a**), diastolic blood pressure (**b**), and heart rate (**c**) in Beagle dogs

As illustrated in Fig. 1, mean systolic/diastolic blood pressure and heart rate showed no statistically significant deviations between the JBD0131-treated groups and concurrent controls (*P* > 0.05). Although minor fluctuations from pre-dose baselines occurred at sporadic intervals, the mean values remained comparable to controls, confirming a lack of toxicological relevance. Comprehensive clinical data for this study are presented in Table S3. Overall, JBD0131 demonstrated no significant cardiovascular toxicity at oral doses up to 300 mg/kg in Beagle dogs.

### Single-dose oral administration shows no significant toxicity in SD rat

This study aimed to determine the maximum tolerated dose (MTD) and identify potential target organs for acute toxicity in SD rats. Rats were administered a single oral dose of 500, 1000, or 2000 mg/kg of JBD0131 and observed for 14 days prior to necropsy. Throughout the 14-day observation period, all SD rats remained in good general condition, with normal activity, clean fur, and no clinical signs of toxicity. As shown in Table S4, body weight and food consumption in both sexes showed no significant differences compared with controls (*P* > 0.05).

On Day 15, male rats in the 500 mg/kg group exhibited statistically significant decreases in NEU and increases in RBC, HGB, and HCT (*P* ≤ 0.05) (Table 2), accompanied by mild elevations in ALB, TP, Urea, and Na^+^; however, these lacked dose-responsiveness and remained within biological variation. At the scheduled necropsy on Day 15, no treatment-related gross abnormalities or significant changes in major organ weights were observed (Table S4)..

**Table 2:** Effects of oral JBD0131 on hematological parameters in male SD rats (Day 15)

Parameter	Control group		Vehicle group		50 mg/kg group		150 mg/kg group		500 mg/kg group	
	n	Mean ± SD	n	Mean ± SD	n	Mean ± SD	n	Mean ± SD	n	Mean ± SD
WBC (10^9^/L)	5	5.12 ± 1.21	5	5.35 ± 0.75	5	5.80 ± 1.36	5	6.53 ± 2.36	5	5.94 ± 2.48
NEU% (%)	5	12.8 ± 3.2	5	9.8 ± 2.4	5	8.5 ± 2.1	5	12.3 ± 3.8	5	11.8 ± 3.8
LYM % (%)	5	83.3 ± 3.4	5	86.4 ± 3.0	5	87.9 ± 2.5	5	83.9 ± 3.5	5	84.7 ± 3.9
MONO% (%)	5	1.4 ± 0.1	5	1.2 ± 0.4	5	1.3 ± 0.4	5	1.5 ± 0.5	5	1.2 ± 0.3
EOS% (%)	5	0.68 ± 0.37	5	0.80 ± 0.20	5	0.60 ± 0.14	5	0.72 ± 0.22	5	0.54 ± 0.23
BASO% (%)	5	0.06 ± 0.09	5	0.12 ± 0.04	5	0.08 ± 0.04	5	0.10 ± 0.00	5	0.02 ± 0.04
NEU (10^9^/L)	5	0.63 ± 0.03	5	0.51 ± 0.11	5	0.48 ± 0.10 *	5	0.74 ± 0.15	5	0.63 ± 0.02
LYM (10^9^/L)	5	4.29 ± 1.14	5	4.64 ± 0.77	5	5.11 ± 1.31	5	5.54 ± 2.16	5	5.11 ± 2.38
MONO (10^9^/L)	5	0.07 ± 0.02	5	0.06 ± 0.01	5	0.08 ± 0.01	5	0.10 ± 0.05	5	0.07 ± 0.05
EOS (10^9^/L)	5	0.032 ± 0.013	5	0.040 ± 0.007	5	0.034 ± 0.005	5	0.042 ± 0.019	5	0.030 ± 0.007
BASO (10^9^/L)	5	0.002 ± 0.004	5	0.004 ± 0.005	5	0.006 ± 0.005	5	0.004 ± 0.005	5	0.002 ± 0.004
RBC (10^12^/L)	5	6.90 ± 0.29	5	6.85 ± 0.25	5	7.62 ± 0.54 *	5	6.72 ± 0.33	5	6.86 ± 0.38
HGB (g/L)	5	139 ± 6	5	140 ± 4	5	149 ± 5 *	5	136 ± 4	5	137 ± 4
HCT (%)	5	42.4 ± 1.7	5	42.4 ± 1.1	5	46.1 ± 1.7 *	5	41.7 ± 1.6	5	41.9 ± 1.3
MCV (fL)	5	61.4 ± 2.1	5	61.9 ± 2.2	5	60.6 ± 2.4	5	62.1 ± 1.3	5	61.2 ± 1.9
MCH (pg)	5	20.1 ± 0.8	5	20.4 ± 0.6	5	19.6 ± 0.8	5	20.3 ± 0.4	5	20.0 ± 0.8
MCHC (g/L)	5	328 ± 4	5	330 ± 3	5	324 ± 3	5	327 ± 2	5	327 ± 8
PLT (10^9^/L)	5	947 ± 49	5	950 ± 61	5	1036 ± 102	5	963 ± 103	5	907 ± 70
RET% (%)	5	3.20 ± 0.43	5	3.02 ± 0.38	5	3.11 ± 0.62	5	3.06 ± 0.27	5	3.12 ± 0.44
RET (10^12^/L)	5	0.220 ± 0.025	5	0.207 ± 0.028	5	0.235 ± 0.039	5	0.205 ± 0.013	5	0.216 ± 0.027
APTT (sec)	5	9.9 ± 2.2	5	9.5 ± 0.7	5	8.7 ± 1.6	5	7.9 ± 1.2	5	9.7 ± 1.2
PT (sec)	5	9.1 ± 0.4	5	9.1 ± 0.5	5	8.9 ± 0.4	5	9.0 ± 0.4	5	9.0 ± 0.4

Neutrophils (NEU) are indicated as NEUT in the Pristima system; lymphocytes (LYM) are indicated as LYMPH; reticulocyte count and percentage (RET and RET%) are indicated as RETIC and %RETIC, respectively. * indicates a statistically significant difference from the control group (*P* ≤ 0.05)

*WBC* White blood cells, *NEU%* neutrophil percentage, *LYM%* lymphocyte percentage, *MONO%* monocyte percentage, *EOS%* eosinophil percentage, *BASO%* basophil percentage, *NEU* neutrophil count, *LYM* lymphocyte count, *MONO* monocyte count, *EOS* eosinophil count, *BASO* basophil count, *RBC* red blood cells, *HGB* hemoglobin, *HCT* hematocrit, *MCV* mean corpuscular volume, *MCH* mean corpuscular hemoglobin, *MCHC* mean corpuscular hemoglobin concentration, *PLT* platelets, *RET%* reticulocyte percentage, *RET* reticulocyte count, *APTT* activated partial thromboplastin time, *PT* prothrombin time

### Single-dose oral administration shows no significant toxicity in Beagle dogs

To evaluate the acute toxicity and toxicokinetic (TK) profile of JBD0131 in a non-rodent species, we performed a single-dose study in Beagle dogs. Dogs received oral doses of 500 or 1000 mg/kg, followed by a 14-day observation and comprehensive TK analysis. While transient vomiting occurred within 2 h of dosing in some animals, no persistent signs of toxicity were observed. Vital signs, electrocardiogram (ECG) parameters, and clinical pathology (hematology and biochemistry) showed no dose-dependent adverse effects (Table S5). TK analysis revealed dose-proportional exposure of JBD0131 and its primary metabolite, DM131. DM131 is formed via the metabolic reduction of the 6-nitro group on the imidazooxazole ring, a process that typically represents a detoxification pathway in mammals. Necropsy confirmed the absence of gross organ abnormalities [17].

Overall, the MTD in Beagle dogs was established at 1000 mg/kg, and the metabolic conversion to DM131 suggests a favorable detoxification mechanism for this class of compounds.

### Repeat-dose oral administration shows no significant toxicity in SD rat

To characterize the sub-chronic toxicological profile and determine the no-observed-adverse-effect level (NOAEL), JBD0131 was administered repeatedly to SD rats. SD rats were orally administered JBD0131 at 30, 120, or 480 mg/kg/day for 4 weeks, followed by a 4-week recovery. No treatment-related changes were observed in clinical signs, body weight, food consumption, ophthalmology, hematology, clinical chemistry, urinalysis parameters, or organ weights (Table S6).

Plasma exposure of JBD0131 and its metabolite DM131 showed no marked sex differences at either the first or last dose. Exposure increased less than proportionally with dose, and no accumulation was detected over 4 weeks. The NOAEL was determined to be 480 mg/kg/day. At this dose, plasma exposures after the last dose were 8680 and 2230 h*ng/mL for JBD0131 and DM131 in females, and 7930 and 2700 h*ng/mL in males (Table 3).

**Table 3:** Mean toxicokinetic parameters of JBD0131 and DM131 in SD rat plasma following oral gavage of JBD0131

Test compound	Days of administration	Sex	Dose (mg/kg)	n	AUC_last_ (h*ng/mL)		C_max_ (ng/mL)	
					**Mean**	**SD**	**Mean**	**SD**
JBD0131	1	Female	30	5	3000	721	251	17.4
			120	5	7890	2130	522	83.9
			480	5	26,300	20,600	802	104
		Male	30	5	3650	255	239	16.1
			120	5	8280	4550	425	18.4
			480	5	18,700	12,100	643	95.4
	28	Female	30	5	3490	440	223	11.5
			120	5	4860	307	358	44.1
			480	5	8680	1600	448	66.3
		Male	30	5	2390	130	174	17.8
			120	5	3640	442	227	20
			480	5	7930	2070	343	32.2
DM131	1	Female	30	5	1210	274	87.6	11.7
			120	5	1050	358	54.5	12.2
			480	5	4960	4260	109	40.1
		Male	30	5	1410	110	90.7	5.8
			120	5	1970	1180	84.4	11.8
			480	5	3130	2480	97.7	30.3
	28	Female	30	5	882	179	50.6	4.71
			120	5	1650	291	94	13.6
			480	5	2230	609	82.1	12.7
		Male	30	5	643	135	40.4	5.64
			120	5	1310	136	57.5	3.24
			480	5	2700	973	88	18.7

AUC_last_area under the plasma concentration–time curve from time zero to the last measurable concentration

C_max_maximum plasma concentration

Overall, the 4-week NOAEL in SD rats was determined to be 480 mg/kg/day, supporting the safety of JBD0131 for extended administration.

### Repeat-dose oral administration shows no significant toxicity in Beagle dogs

A 4-week repeat-dose study was conducted in Beagle dogs to assess sub-chronic toxicity and potential species-specific sensitivities. Beagle dogs were orally administered JBD0131 at 15, 60, or 300 mg/kg/day for 4 weeks, followed by a 4-week recovery. In the 60 and 300 mg/kg male groups, lead II ECG showed a slight trend toward QT interval prolongation (Table S7.8). Otherwise, no treatment-related changes were observed in clinical signs, body weight, food consumption, ophthalmology, blood pressure, body temperature, ECG parameters, hematology, clinical chemistry, urinalysis, or organ weights in either sex (Table S7).

Plasma exposures of JBD0131 and its metabolite DM131 showed no marked sex differences after the first or last dose. While no accumulation of the parent compound JBD0131 was observed over the 4-week administration period, the metabolite DM131 showed moderate accumulation (Table S7). This mechanistic interpretation is reinforced by our first-in-human study, which demonstrated that systemic exposure to DM131 was well-tolerated and not associated with any dose-limiting toxicities in healthy adults [15].

The NOAEL was determined to be 300 mg/kg/day for females and 15 mg/kg/day for males. This sex-based discrepancy is primarily attributed to a slight trend toward QT interval prolongation observed exclusively in males at doses of 60 and 300 mg/kg/day, whereas females exhibited no such cardiovascular sensitivity even at the highest exposure level. Specifically, at 300 mg/kg/day, males exhibited a mean QTc increase of approximately 27 ms (~ 10%) from baseline. At these respective NOAEL doses, the plasma exposures (AUC_last_) after the final dose were 30,300 and 3630 h*ng/mL in females, and 7180 and 1430 h*ng/mL in males for JBD0131 and DM131, respectively (Table 4).

**Table 4:** Exposure of JBD0131 and DM131 in Plasma of Beagle Dogs Following Oral Gavage Administration of JBD0131

Analyte	Dosing days	Sex	Dose (mg/kg)	n	AUC_0–24_ (h*ng/mL)		AUC_0–96_ (h*ng/mL)	
					Mean	SD	Mean	SD
JBD0131	1	Female	15	5	5020	1600	/	/
			60	5	10,700	2800	/	/
			300	5	12,900	4950	/	/
		Male	15	4*	5570	1900	/	/
			60	5	13,900	4120	/	/
			300	5	20,000	4270	/	/
	28	Female	15	5	7340	2510	8530	3690
			60	4#	15,700	3840	24,800	9280
			300	5	19,400	5270	30,300	7150
		Male	15	5	6530	1930	7180	2820
			60	5	14,900	1520	20,100	3350
			300	5	22,200	4870	32,300	8390
DM131	1	Female	15	5	581	190	/	/
			60	5	550	107	/	/
			300	5	767	248	/	/
		Male	15	4*	630	192	/	/
			60	5	721	148	/	/
			300	5	1610	464	/	/
	28	Female	15	5	1190	363	1640	559
			60	4#	1720	656	3470	1680
			300	5	1850	489	3630	975
		Male	15	5	1090	363	1430	486
			60	5	1780	381	3100	977
			300	5	2270	694	4560	1620

^*^Following the first dose, the plasma concentration of Dog No. 3M002 in the 15 mg/kg group was significantly lower than that of other animals in the same group; therefore, its TK parameters were excluded from the statistical analysis

^#^Following the final dose, Dog No. 4F003 in the 60 mg/kg group vomited within 20 min after administration; therefore, its TK parameters were excluded from the statistical analysis

Overall, repeated oral administration of JBD0131 was generally well tolerated in Beagle dogs, with minor ECG changes at higher doses in males as the only observed effect. Given that these transient QTc fluctuations occurred only at supra-therapeutic exposures (300 mg/kg/day) and were not associated with adverse clinical symptoms, the implications for human risk at intended therapeutic dose levels are considered minimal. Nevertheless, this preclinical signal establishes a critical evidence-based rationale for the inclusion of rigorous cardiovascular monitoring in subsequent clinical development phases, ensuring that any potential class-related cardiac liabilities are proactively managed in patients.

## Discussion

The comprehensive preclinical evaluation of JBD0131, a novel nitroimidazooxazole derivative, reveals a highly favorable and predictable safety profile. The fundamental principle underlying these findings is the existence of a wide therapeutic window between effective therapeutic exposure and the onset of systemic toxicity. Repeated-dose studies in SD rats showed no treatment-related mortality, which is a significant finding given that the nitroimidazooxazole class can sometimes exhibit off-target effects at high doses. The observation that minimal changes in clinical chemistry or hematology were incidental and non-adverse, along with the absence of delayed effects post-recovery, underscores the compound's metabolic stability and low intrinsic toxicity.

JBD0131 represents a significant breakthrough in the quest for safer antitubercular agents, particularly when compared to existing clinical standards. While second-line drugs like bedaquiline and pretomanid have significantly improved treatment outcomes, their clinical use is often tempered by concerns regarding hepatotoxicity and QTc prolongation [18–20]. Our data suggests that JBD0131 possesses a more favorable preclinical safety profile in the models tested than historically reported for these agents.

A critical discovery in our study was the sex-specific sensitivity observed in the dog model. The NOAEL was established at 15 mg/kg for males and 300 mg/kg for females. Despite comparable systemic exposures between sexes, male dogs exhibited a slight trend toward QTc prolongation at higher doses, a trait observed in other compounds of this chemical class. However, the magnitude of these changes was minor and non-adverse, occurring at exposures far exceeding the predicted therapeutic range, which suggests a superior cardiac safety margin compared to bedaquiline. Furthermore, The slight trend toward QTc prolongation in male dogs was considered a primary electrophysiological effect rather than secondary to structural cardiac changes, as supported by the normal heart weights and heart-to-body weight ratios.

The established NOAEL of 480 mg/kg/day in rats provided a robust toxicological foundation for determining the initial Human Equivalent Dose, effectively facilitating the clinical entry of JBD0131. Notably, the translational validity of these preclinical findings has been convincingly substantiated by the successful completion and subsequent publication of Phase I and Phase II clinical trials [15, 16].

The integration of our toxicological thresholds with the observed linear and predictable pharmacokinetics—characterized by stable clearance and a lack of systemic accumulation—accurately forecasted the drug's behavior in humans. The favorable safety profiles and therapeutic efficacy reported in clinical cohorts align closely with the wide therapeutic window identified in our animal models. This consistency confirms that the 4-week repeated-dose toxicity and safety pharmacology assessments provided a highly reliable prediction of JBD0131’s clinical safety margin, bridging the gap between bench research and successful clinical application.

Despite the significant breakthroughs, several limitations of this manuscript warrant consideration. First, while the current 4-week study duration supported early-phase trials, it may not fully account for late-onset or cumulative toxicities that could emerge during the extended treatment regimens (typically 6 months or longer) required for tuberculosis therapy. Therefore, future 13-week or 26-week chronic toxicity studies remain essential to satisfy regulatory requirements for long-term administration. Second, additional evaluations concerning reproductive and developmental toxicity (DART) and genotoxicity are necessary to complete the comprehensive safety profile required for late-stage development. Finally, the comparisons between JBD0131 and existing agents like bedaquiline or pretomanid are based on indirect cross-study literature reviews. Given the inherent variations in experimental conditions across different laboratories, these observations should be considered qualitative until confirmed by direct head-to-head comparative trials.

In summary, the comprehensive toxicological and pharmacological profiling of JBD0131 demonstrates a robust safety margin and highly predictable pharmacokinetic behavior. The translational relevance of these preclinical findings has been successfully substantiated by the published Phase I and Phase II clinical outcomes. While direct head-to-head comparisons are required for definitive confirmation, the observed safety profile of JBD0131—particularly regarding cardiac repolarization—compares favorably with literature-reported data for existing second-line agents like bedaquiline and pretomanid. In conclusion, JBD0131 demonstrated a favorable safety profile characterized by the absence of treatment-related mortality and the maintenance of normal physiological and organ weight parameters at supra-therapeutic doses.

## Materials and methods

### Chemicals and formulations

JBD0131, a novel and potent nitroimidazooxazole derivative designed and screened by our group, was synthesized by Changzhou Yinsheng Pharmaceutical Co., Ltd. (Changzhou, China) [13]. Accurately weigh the required amount of JBD0131 into a mortar. Add Kolliphor HS15 (Sigma) to achieve a final concentration of 10% (v/v) of the total volume and grind thoroughly. Subsequently, add PEG400 (Chengdu Kelong Chemical Reagent Factory) to achieve a final concentration of 20% (v/v) of the total volume, and continue grinding until a flowable mixture is obtained. Transfer the mixture into a pre-calibrated container. Rinse the mortar with an appropriate volume of sterile water for injection, and transfer the rinsing solution into the same container. Adjust the volume to the calibration mark with sterile water for injection, and sonicate in a water bath for 10 min.

### Animals

Sprague–Dawley (SD) rats (5–6 weeks old; females: 181.6–212.6 g, males: 205.6–346.4 g) were obtained from Beijing Vital River Laboratory Animal Technology Co., Ltd. (Beijing, China) and housed in a Specific Pathogen-Free (SPF) facility. Conventional Beagle dogs (15.5–16.5 months old; 8.20–10.40 kg) were purchased from Beijing Mars Biotechnology Co., Ltd. (Beijing, China) and maintained in a conventional animal room. All animal experiments, including the housing of Beagle dogs, were conducted at the animal facilities of Chengdu Huaxi Haichi Pharmaceutical Technology Co., Ltd. (Chengdu, China).

All animal experiments in this study were commissioned to and professionally executed by the technical staff at Chengdu Huaxi Haichi Pharmaceutical Technology Co., Ltd. (Chengdu, China). Specifically, the in vivo administration, clinical observations, and sample collection were performed by the facility's qualified personnel in accordance with their standard operating procedures (SOPs).

### Functional observational battery (FOB)

FOB assessments were performed to evaluate the potential neurobehavioral effects of JBD0131. Male and female SD rats were used in the assay. Based on available experimental data, rats were randomly assigned to three dose groups (50, 150, and 500 mg/kg, representing low, medium, and high doses, respectively) and administered orally at 5 mL/kg. Equal volumes of vehicle solutions containing 10% (v/v) Solutol and 20% (v/v) PEG400 in water, or sterile injection water, were used as control.

The evaluations included home-cage observations (e.g., spontaneous locomotion, grooming, and aggression), responses during handling/restraint, open-field activity (locomotion, rearing, and urination), stimulus–response tests (visual, auditory, and tactile reflexes), as well as measurements of grip strength and body temperature.

Behavioral parameters such as urination and rearing counts were recorded at approximately 8, 24, and 48 h after administration and expressed relative to either the pre-dose baseline or the corresponding control group.

### Effects of JBD0131 on respiratory function in SD rats

For the respiratory function assessment, the dosing route and procedures were identical to those described for the FOB test. Before dosing, each animal was placed into a whole-body plethysmography chamber for at least 0.5 h, and a stable segment of the recording was selected as the pre-dose baseline. For each scheduled measurement, rats were placed in the plethysmography chamber, and respiratory signals were recorded continuously for at least 10 min at that time point.

Respiratory parameters—including respiratory rate (BPM, breaths per minute), tidal volume (TV, mL), and minute ventilation (MV, mL/min)—were measured once before dosing and at approximately 8 h (± 30 min), 24 h (± 60 min), and 48 h (± 60 min) after dosing.

### Cardiovascular safety assessment of JBD0131 in Beagle dogs

Based on preliminary studies, low, medium, and high oral dose levels of JBD0131 were set at 15, 60, and 300 mg/kg, respectively. The selection of these doses was based on two internal preliminary repeat-dose studies in Beagle dogs. First, in a 4-week study at doses of 15, 30, and 100 mg/kg, dose-related vomiting and loose stools were observed, with slight QTc prolongation noted at 100 mg/kg. Second, in a 14-day escalation study at 15, 60, and 300 mg/kg, JBD0131 was generally well-tolerated, with only occasional unformed stools at 300 mg/kg by Day 14. Thus, 300 mg/kg was selected as the high dose for the current 4-week study to further evaluate its long-term impact on cardiac repolarization and to define the toxicological ceiling. Equal volumes of vehicle or saline were used as control treatments. Beagle dogs (*n* = 6; females = 3, males = 3) received the test article according to a 6 × 5 modified Latin square design (Table S8).

Cardiovascular parameters were monitored using an implantable wireless telemetry system (DSI, USA). Prior to the first administration, continuous recordings of systolic blood pressure, diastolic blood pressure, mean arterial pressure, and lead II electrocardiographic signals were obtained for approximately 22 h to establish baseline values. For each dosing period, electrocardiogram (ECG parameters) and blood pressure were recorded continuously for at least 60 min before dosing and for at least 48 h post-dose.

### Single-dose toxicity

Single-dose toxicity studies were conducted in both SD rats and Beagle dogs. For SD rats, low-, mid-, and high-dose groups of JBD0131 were set at 500, 1000, and 2000 mg/kg, respectively. For Beagle dogs, low- and high-dose groups were set at 500 and 1000 mg/kg. Equal volumes of vehicle or saline were used as control treatments.

Both species received a single oral dose on Day 0 and were monitored for a 14-day observation period. Evaluated parameters included food consumption, body weight, hematology, serum biochemistry, and gross necropsy findings. In Beagle dogs, additional assessments included lead II electrocardiography and blood pressure monitoring, urinalysis, and toxicokinetic analysis.

### Repeat-dose toxicity

Repeat-dose toxicity studies were performed in both SD rats and Beagle dogs. For SD rats, the low-, mid-, and high-dose groups of JBD0131 were set at 30, 120, and 480 mg/kg, respectively. For Beagle dogs, the corresponding dose levels were 15, 60, and 300 mg/kg. Equal volumes of vehicle or saline were administered to the control groups.

All animals received once-daily oral gavage for 4 consecutive weeks, followed by a 4-week recovery period. The first day of dosing was designated as Day 1, and the day after the final administration marked the beginning of the recovery phase.

During the study, comprehensive toxicological parameters were monitored, including body weight, food consumption, body temperature, blood pressure, electrocardiography, hematology, clinical biochemistry, urinalysis, and bone marrow smear evaluation examination.

### Statistical analysis

Quantitative variables (e.g., body weight and food consumption) were expressed as mean ± SD, and qualitative variables (binary, ordinal, or nominal) were described using frequency counts.

For the FOB safety evaluation, quantitative variables were analyzed using repeated-measures ANOVA. Multiple comparisons among groups at each time point were performed using multivariate ANOVA followed by Dunnett’s test. Within-group comparisons across time points were conducted using repeated-measures ANOVA followed by the Least Significant Difference (LSD) test.

For toxicity studies, homogeneity of variance was first assessed using Levene’s test. If variances were homogeneous, one-way ANOVA was performed; otherwise, the Kruskal–Wallis H test was applied. When ANOVA showed significance (*P* ≤ 0.05), Dunnett’s test was used for post-hoc comparison. When the Kruskal–Wallis H test was significant (*P* ≤ 0.05), group differences were analyzed using the Mann–Whitney U test. Ordinal categorical variables were analyzed using the Kruskal–Wallis H test, and if significant, followed by the Mann–Whitney U test. Binary or nominal categorical variables were analyzed using Fisher’s exact test, and when results were significant (*P* ≤ 0.05), pairwise comparisons were also conducted using Fisher’s exact test. Body weight, food consumption, hematology, clinical chemistry, and organ weight data were analyzed using the PRISTIMA 6.1.1 data acquisition system.

All tests were two-sided with α = 0.05. Statistical analyses were performed using SPSS 13.0 for Windows. To maintain the family-wise error rate and minimize false-positive findings across multiple endpoints, Dunnett’s test was strictly applied for post-hoc comparisons following ANOVA. For repeated measurements, the potential for multiplicity was further controlled by analyzing time-dependent trends rather than isolated time-point deviations.

Findings were characterized as 'non-adverse' if they met the following criteria: (1) the magnitude of change was within the laboratory’s historical control range; (2) the change lacked a clear dose–response relationship; (3) the finding was not associated with secondary alterations in related clinical chemistry; and (4) the change showed evidence of reversibility.

## Supplementary Information

Supplementary Material 1.

## Data Availability

Data generated or analyzed during this study are included in this published article (and its supplementary information files).
